# Chemical strategies for the covalent modification of filamentous phage

**DOI:** 10.3389/fmicb.2014.00734

**Published:** 2014-12-23

**Authors:** Jenna M. L. Bernard, Matthew B. Francis

**Affiliations:** ^1^Department of Chemistry, University of California at BerkeleyBerkeley, CA, USA; ^2^Materials Sciences Division – Lawrence Berkeley National LaboratoriesBerkeley, CA, USA

**Keywords:** filamentous phage, chemical modification, bioconjugation, protein modification, polymers, delivery

## Abstract

Historically filamentous bacteriophage have been known to be the workhorse of phage display due to their ability to link genotype to phenotype. More recently, the filamentous phage scaffold has proven to be powerful outside the realm of phage display technology in fields such as molecular imaging, cancer research and materials, and vaccine development. The ability of the virion to serve as a platform for a variety of applications heavily relies on the functionalization of the phage coat proteins with a wide variety of functionalities. Genetic modification of the coat proteins has been the most widely used strategy for functionalizing the virion; however, complementary chemical modification strategies can help to diversify the range of materials that can be developed. This review emphasizes the recent advances that have been made in the chemical modification of filamentous phage as well as some of the challenges that are involved in functionalizing the virion.

## INTRODUCTION

**FIGURE 1:**
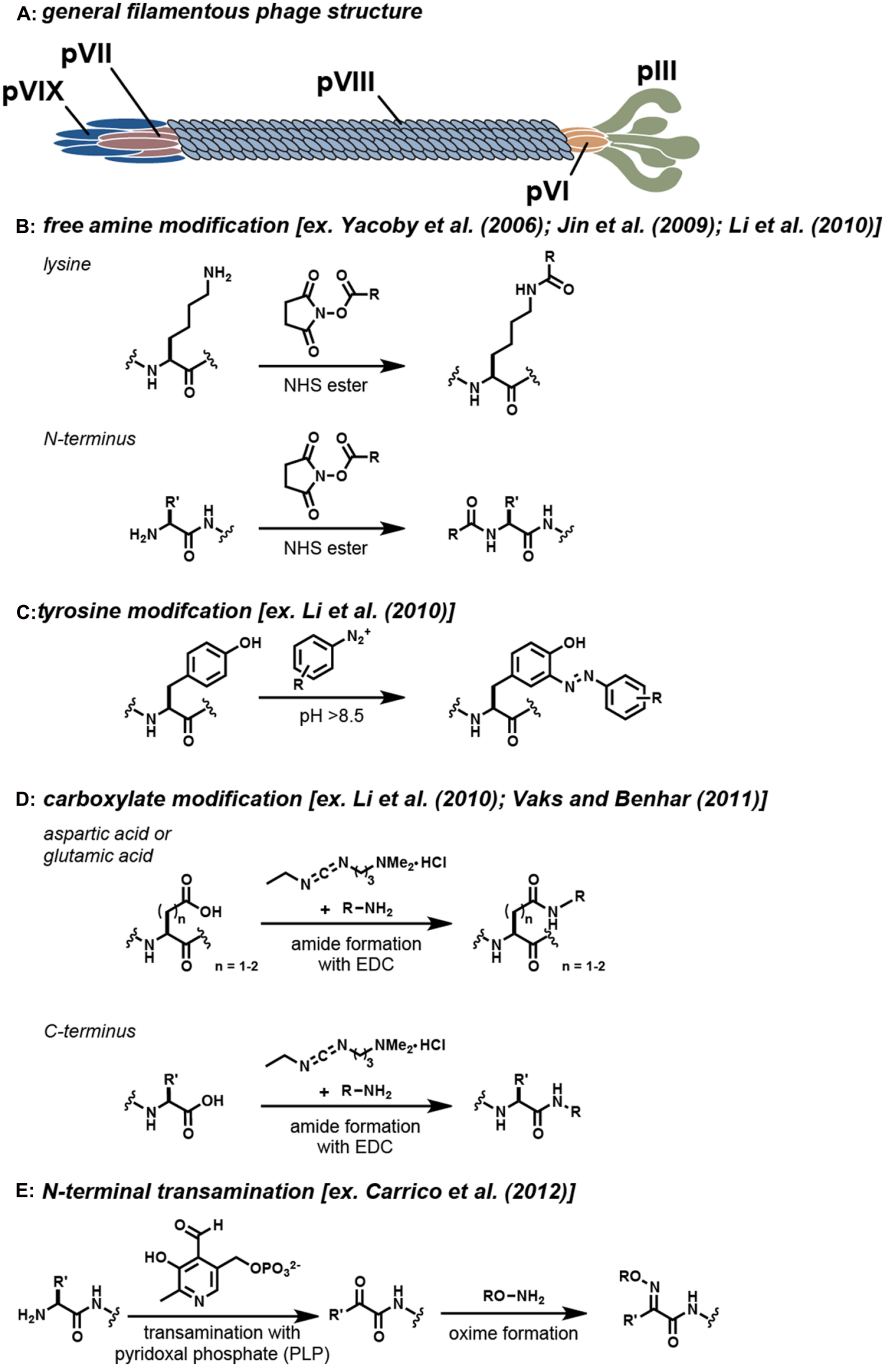
**Covalent modification strategies for filamentous phage. (A)** The general locations of the coat proteins are shown. Note that actual phage have significantly more copies of the pVIII protein than are shown. Typical reactions that are used to modify specific functional groups on these proteins are shown in **(B–E)**. For each strategy, example references appear in the brackets.

The Ff group of filamentous bacteriophage are non-lytic viruses that infect bacteria by attachment to their F pili (Smith and Petrenko, 1997; Kehoe and Kay, 2005). M13, f1, and fd are the most well-characterized types of this virus and have a DNA sequence similarity up to 98.5% (Rakonjac et al., 2011). These viruses are known to be the workhorses of phage display, as they can be selected and evolved to identify novel binding activity through repeated cycles of mutation, selection, and amplification. This methodology allows for the production of phage constructs that can bind a variety of targets, including proteins, polymers, small molecules, and metal ions (Smith and Petrenko, 1997; Kehoe and Kay, 2005). In terms of structure, filamentous phage exist as long, thin tubes with a large aspect ratio (∼900 nm in length and 6 nm in width). The capsids comprise of five copies of each minor coat protein (pIII, pVI, pVII, and pIX) and a varying amount of the major coat protein (pVIII; Li et al., 2010). The interior of the virion contains a single-stranded deoxyribonucleic acid (ssDNA) genome that is protected by the cylindrical protein housing formed by the pVIII subunits. The minor coat proteins form caps at both ends of the assembly and are involved in host infection. The overall filamentous phage structure is shown in **Figure 1A**.

In addition to their important role in molecular evolution, filamentous phage have served as attractive platforms for a wide variety of applications due to their stability, ability to display peptides with strong affinities for their targets, and ease of production by propagation in bacteria. Moreover, the high number of repetitive coat proteins in the virion allow for multiple copies of a desired functionality to be displayed with well-defined spatial relationships. Reported applications include light-harvesting (Nam et al., 2010), nanoparticle nucleation (Mao et al., 2004), electrode templating (Lee et al., 2009), cell growth and differentiation (Merzlyak et al., 2009), bactericidal agents (Hagens and Blasi, 2003), molecular imaging (Hajitou et al., 2006; Chen et al., 2007; Li et al., 2010; Carrico et al., 2012; Ghosh et al., 2012), and drug delivery (Bar et al., 2008; Suthiwangcharoen et al., 2011; Ghosh et al., 2012).

The use of these virions in many applications requires the integration of targeting groups and/or cargo molecules that cannot be biosynthesized. For example, early work used phage display to evolve M13 phage coat proteins to bind semiconductors of varied compositions and geometries (Whaley et al., 2000). Continued work in this field has used similar techniques to develop phage-based scaffolds that can serve as improved anodes in lithium ion batteries, leading to enhanced storage capacity, improved conductivity, and the ability to produce thin flexible 2D arrays (Lee et al., 2002; Nam et al., 2006; Avery et al., 2009). In addition, applications in the fields of drug delivery and medical imaging have also relied heavily on the functionalization of filamentous phage particles with a range of synthetic molecules (Deutscher and Kelly, 2011).

In early work, the genetic manipulation of the phage coat proteins to incorporate peptides with novel function was the standard approach for filamentous phage modification (Willats, 2002). More recently, bioconjugation techniques have been exploited to allow a greater variety of synthetic molecules to be attached to the phage. The incorporation of these bioconjugation techniques has allowed for the production of a much more diverse set of constructs. As examples, the phage scaffold has been successfully modified with a variety of small organic molecules (Niu et al., 2008; Carrico et al., 2012), quantum dots (Merzlyak et al., 2009), fluorophores (Goldman et al., 2000; Hilderbrand et al., 2005, 2008; Carrico et al., 2012), and drugs (Yacoby et al., 2006; Bar et al., 2008). Despite these accomplishments, however, there still exist a limited number of chemical labeling strategies to modify the filamentous phage coat proteins. The most widely used strategies rely on reagents that target reactive amino acid residues present in the sequences (i.e., the N-terminus, lysines, cysteines, tyrosines, aspartic acids, and glutamic acids). A summary of the strategies that have been frequently used to modify the virion is shown in **Figure 1**. As powerful as these modification strategies are, the majority of these techniques lack the high degree of specificity and control that is needed for many applications. The ideal bioconjugation reaction should be able to modify targets in a site-specific manner while still conserving the integrity of the construct by operating in aqueous media under mild pH and temperature conditions.

With these considerations in mind, this review will focus on the recent chemical modification strategies that have been implemented on filamentous phage for materials construction. It should be noted that several detailed discussions regarding the use of filamentous phage in the context of phage display (Smith and Petrenko, 1997; Kehoe and Kay, 2005), gene therapy (Verma and Weitzman, 2005; Schaffer et al., 2008), and adjuvants for vaccines (Garcea and Gissmann, 2004) have appeared, and thus these applications will not be emphasized in this review. In addition, several in-depth reviews on the structure, replication cycle, and general applications of the filamentous phage have been also published elsewhere (Hemminga et al., 2010; Branston et al., 2011; Rakonjac et al., 2011).

## MAIN

### GENETIC MODIFICATION OF THE COAT PROTEINS

The most widely used strategy for functionalizing the phage scaffold has been the genetic incorporation of peptides that are engineered to associate with a novel target or serve as reactive handles (Rakonjac et al., 2011). It has been demonstrated that each type of coat protein present on the filamentous phage possesses the ability to display foreign proteins through genetic modification (Kwasnikowski et al., 2005; Smelyanski and Gershoni, 2011; Ghosh et al., 2012). The minor pIII coat protein is the most widely used to display protein motifs due to its ability to tolerate a high number and variety of amino acid insertions (Kwasnikowski et al., 2005). In applications where a large loading capacity is desired, such as drug delivery or imaging, it is common to label the major coat protein, pVIII, due to its high copy number (approximately 2700 monomers for M13 and f1, and 4,200 monomers for fd). However, it is important to note that genetic engineering of this major coat protein is less common because the pVIII monomers can only tolerate the incorporation of 6–8 amino acids (Popp et al., 2007; Hess et al., 2012).

With the incorporation of peptide sequences onto the phage scaffold comes the ability to modify the phage using either enzyme-based ligation techniques or chemoselective bioconjugation reactions. The genetic incorporation of enzymatic ligation handles has been demonstrated in several phage systems, including the biotin ligase (Chen et al., 2007; Smelyanski and Gershoni, 2011) and sortase A (Hess et al., 2012) systems. These methods are particularly useful in terms of their high degree of specificity compared to chemical approaches. Alternatively, several examples that use genetic modification to incorporate an amino acid that can act as a functional handle also exist. For example, genetically incorporated cysteine residues have been shown to be modified through native chemical ligation (Dwyer et al., 2000), thiol alkylation (Jespers et al., 2004; Heinis et al., 2009), and maleimide modification (Santoso et al., 2013). However, with these latter strategies, one has to take great care in the placement of the cysteine residues to limit problems with disulfide cross-linking between the closely packed phage coat proteins. Furthermore, the chemical modification of introduced cysteines can result in the unwanted labeling of endogenous solvent exposed cysteines found on the phage coat proteins (pVI, pVII, pIX; Soumillion et al., 1994; Pedersen et al., 1998). One way to circumvent these difficulties is to use immobilized tris(2-carboxyethyl)phosphine (iTCEP) to reduce only the sterically accessible cysteines on a peptide sequence displayed on the pIII coat protein of the phage (Jafari et al., 2014). Using this modification strategy, the Derda group was able to create a light-inhibited ligand system by alkylating and reducing two cysteines on the pIII that flank a randomized heptamer peptide sequence that was incorporated into the phage by phage display. Although the genetic incorporation of amino acids has proven to be a powerful tool for installing peptide motifs and natural amino acids as functional handles on the pIII protein, this strategy is limited to a low number of modifications on phage if the major coat protein cannot tolerate these genetic insertions following the signal sequence.

### FREE AMINE MODIFICATION

One of the most widely used strategies for the chemical modification of the filamentous phage relies on the non-specific modification of the free amine groups of the phage coat proteins. A number of strategies exist to target amines on proteins, including the use of isothiocyanates, isocyanates, acyl azides, and *N*-hydroxysuccinimide (NHS) esters (Hermanson, 2013). Of these, the acylation of amines using NHS esters has seen the most use. This reaction proceeds within an ideal pH range of 7.0–9.2 and results in the formation of a stable amide linkage following NHS release (**Figure 1B**; Mujumdar et al., 1993). Reports by several research groups have demonstrated that NHS ester chemistry is a reliable strategy for modifying the virion coat proteins (**Table 1**), although this approach can also lead to unwanted acylation of the lysine residues on protein fusions. For instance, Yacoby et al. (2006) demonstrated that both fd and M13 phage could serve as targeted drug carriers. In this interesting study, the phage scaffold was first modified genetically to include either a targeting peptide on the pVIII coat protein or a fUSE5-ZZ IgG binding domain on the pIII coat protein. Subsequently, the lysine residues were functionalized with chloramphenicol glutarate derivatives bearing additional NHS ester groups for phage attachment. It was then demonstrated that the phage-chloramphenicol constructs acted as prodrugs because they did not have cytotoxic activity until the labile ester linkages were released at the target site. Although the protein modification strategy proved to be successful overall, several complications arose upon using free amine modification to create this prodrug phage construct. First, the researchers had to take the precaution of selecting the targeting peptide displayed on pVIII such that it did not contain a free lysine in its motif. Additionally, the fuSE5-ZZ binding moiety displayed on the pIII protein had to be protected with human protein A-purified IgG prior to being conjugated with the chloramphenicol derivatives. Another difficulty that the researchers faced was that the high percentage of modification of the phage proteins caused them to become unstable and insoluble.

**Table 1:** Functionalization of filamentous phage for application.

Application	Modification strategies [G]: Genetic [C]: Chemical	Functionalization	Reference
Protein engineering	[G] Peptide (pIII, pVIII)	Native chemical ligation	Dwyer et al. (2000)
Combinatorial library	[G] Yeast acceptor peptide (pIII)	Streptavidin-fluorophore	Chen et al. (2007)
Imaging	[G] Sortase motif (pIII, pVIII)	Single domain antibody, fluorescent peptide	Hess et al. (2012)
Immunoassays	[G] Antigen binding site (pIII)[C] Thiol alkylation	Fluorophore	Jespers et al. (2004)
Combinatorial library	[G] Peptide motif (pIII)[C] Thiol alkylation	Small molecule	Heinis et al. (2009)
Light-responsive ligands	[G] Cysteine containing peptide (pIII)[C] Alkylation/reduction	Photoresponsive azobenzene core	Jafari et al. (2014)
Targeted binding	[G] Cysteine containing peptide (pIII)[C] Maleimide	EGFR-binding peptide	Santoso et al. (2013)
Ligand identification	[G] N-terminal Ser/Thr (pIII)[C] Sodium periodate/oxime form.	Glycan	Ng et al. (2012)
Biosensor	[G] Targeting peptide (pIII)[C] NHS ester	Cy5 dye	Goldman et al. (2000)
Imaging application	[G] Targeting peptide (pIII, pVIII)[C] NHS ester	Fluorochromes	Hilderbrand et al. (2005)
Targeted drug delivery	[G] Targeting peptide (pIII, pVIII)[C] NHS ester	Chloramphenicol	Yacoby et al. (2006)
pH-responsive imaging	[C] NHS ester	pH responsive dye	Hilderbrand et al. (2008)
Composite fibers	[C] Lysine modification	Glutaric anhydride	Niu et al. (2008)
NHS kinetic study	[C] NHS ester	AF680	Jin et al. (2009)
Cancer cell imaging	[C] NHS ester, diazonium coupling	Fluorescein, folate azide	Li et al. (2010)
Drug delivery	[C] NHS ester	Folic acid	Suthiwangcharoen et al. (2011)
Cancer cell imaging	[G] scFv (pIII)[C] N-terminal transamination	Small molecule, fluorophore	Carrico et al. (2012)
NMR biosensor	[G] scFv (pIII) [C] N-terminal transamination	^129^Xe cryptophane-A	Palaniappan et al. (2013)

To gain an improved understanding of phage coat protein modification using NHS esters, Jin et al. (2009) developed an empirical kinetic model to predict the modification level of filamentous phage under a wide range of reaction conditions. This study found that fifty percent of the phage maximum binding capacity to streptavidin was reached when approximately 0.03 biotins were attached per pVIII subunit. Such information can reduce the need for over modifying a targeting construct in future studies, perhaps allowing additional modifications to be used to append secondary cargo and/or solubilizing moieties.

Subsequent studies by Li et al. (2010) screened the reactivity of the amino groups of the virion by varying the concentration of *N*,*N*,*N*′,*N*′-tetramethylrhodamine (TMR) NHS ester in a 24 h reaction. Through use of UV-visible spectroscopy, it was found that 1600 rhodamine units could be attached to the M13 bacteriophage, but fluorescence quenching occurred when the phage particles bore more than 400 dyes each. In addition, the study elucidated that some of the six amines present on the M13 pVIII protein (N-terminal alanine 1 and lysines 8, 40, 43, 44, and 48) were more reactive than others. At low levels of modification, Ala-1 was primarily modified and at high modification levels both Ala-1 and Lys-8 were modified.

As another example of using NHS esters to functionalize phage, Li et al. (2010) created a construct for drug delivery using an M13 particle assembly. This structure was functionalized to bind cancer cells by using NHS ester chemistry to attach folic acid. The group was able to demonstrate that each pVIII subunit contained only one modification, and that these assemblies were amenable to loading with doxorubicin. Moreover, it was demonstrated that these constructs were stable under physiological conditions, but disassembled upon being delivered to the target site. This construct is just one of many examples that demonstrate that free amine modification utilizing NHS chemistry is a viable strategy for the creation of functionalized phage (See **Table 1**).

### TYROSINE MODIFICATION

Diazonium groups have long been known to react with a lysine, tyrosine, and histidine residues (Higgins and Harrington, 1959). These highly reactive diazonium groups can be used to modify tyrosine residues at pH values that are typically greater than 8.5 through an electrophilic attack on the active pi system of tyrosine (**Figure 1C**; Hermanson, 2013). The ability of diazonium salts to modify the phage major coat proteins was tested by Li et al. (2010). It was found that this strategy could append approximately 400 biotin groups per phage particle. Modifications occurred on the two tyrosine residues displayed on the pVIII major coat proteins (Tyr 21 and Tyr 24), whereas the lysine and histidine residues remained unmodified. This study is an important demonstration of a chemical modification strategy that can be used to complement the more commonly used lysine modification strategies. Experimental results from this study demonstrated that lower levels of modifications were achieved with the use of diazonium salt modification (400 molecules per phage) instead of NHS ester modification (1600 molecules per phage). It should be noted that this modification strategy non-specifically labels tyrosine moieties on the entire phage scaffold and therefore may not be ideal in scenarios in which binding moieties contain tyrosines. The generation of diazonium salts with nitrous acid also places some limits on the functional groups that can be present on the group to be attached.

### CARBOXYLIC ACID MODIFICATION

Carbodiimide compounds, such as 1-ethyl-3-(-3-dimethylaminopropyl) carbodiimide hydrochloride (EDC) and *N*′,*N*′-dicyclohexyl carbodiimide (DCC), are common bioconjugation reagents used to activate carboxylic acid containing residues. These compounds react with aspartate and glutamate residues to form *O*-acylisourea intermediates, which subsequently react with primary amines to form stable amide bonds, **Figure 1D** (Hermanson, 2013). The reactivity of the carboxylic acid containing residues with carbodiimide chemistry was investigated by Li et al. (2010). In this study, varying concentrations of rhodamine B amine (RB), 1-ethyl-3-(3-dimethylaminopropyl) carbodiimide (EDC), and *N*-hydroxysulfosuccinimide (sulfo-NHS) were allowed to react with the phage coat proteins during a 12 h incubation period. It was found that the RB dye had a low degree of reactivity, and therefore a small molecule analog, propargylamine, was used to identify the reactive moieties. Of the four available carboxylic acid containing sites, only two sites (Glu-2 and Asp4/Asp5) on the pVIII monomers were found to be modified. The second modification site could not be distinguished between Asp4/Asp5 due to their close proximity.

Successive studies done by Vaks and Benhar (2011), demonstrated the capability of EDC coupling to functionalize the phage scaffold as an anti-microbial agent. In this study, the filamentous phage scaffold was covalently attached to chloramphenicol with an aminoglycoside linker using EDC coupling. These modified phage particles were shown to have prodrug qualities, longer circulation times, and lower immunogenicity compared to wild type phage.

The studies described above demonstrate that EDC coupling is a modification strategy that can be used effectively to target the carboxylic acid moieties on the phage scaffold to create new phage materials. As shown by Li et al. (2010), these carboxylic acid moieties are less chemically reactive than their lysine and tyrosine amino acid counterparts, and so are likely to result in lower percent modification of the phage scaffold. Because EDC coupling is not a site-specific chemical modification strategy, any carboxyl entity on a displayed protein is susceptible to modification.

### N-TERMINAL MODIFICATION

In an effort to move toward a site-selective modification approach, an N-terminal transamination modification strategy has been developed using pyridoxal 5′-phosphate (PLP) to functionalize the phage (Carrico et al., 2012). This method involves a two-step site-selective transamination/oxime-formation reaction, wherein the N-terminal amine is transformed into a pyruvamide group that is then converted into an oxime using alkoxyamine reagents (**Figure 1E**). Through this chemistry, 80% of the pVIII N-termini along the capsids could be converted to ketone functional groups, as determined by HPLC. The reaction was found to be selective for only solvent accessible N-terminal amines, with no participation from the ε-amine groups of lysine (Scheck et al., 2008). With this chemistry, fd phage bearing single-chain antibody fragment (scFv) groups previously determined to bind epidermal growth factor receptor (EGFR; Heitner et al., 2001) and human EGFR 2 (HER2; Poul et al., 2000) were modified with fluorophores and polyethylene glycol 2k (PEG2k) to create phage constructs that could distinguish breast cancer cell markers using fluorescence microscopy. Following this initial report, Palaniappan et al. (2013) further explored the versatility of this chemistry to create phage constructs for use as targeted ^129^Xe NMR biosensors. The use of N-terminal transamination/oxime-formation for the functionalization of the phage scaffold provides one of the first examples of a site-selective method that has been used to modify the phage coat proteins. This strategy provides a way to reach high levels of modification while preserving the binding capabilities of peptides that are displayed in other positions. Through the use of strategies such as these, well-defined constructs with multiple functionalities can be obtained.

As an alternative, serine and threonine residues have also been incorporated as latent N-terminal functional handles. The Derda group has demonstrated the functionalization of the virion through genetic introduction of a serine or threonine at the N-terminus of the minor coat pIII proteins (Ng et al., 2012). These phage were treated with sodium periodate to provide aldehydes, allowing for the incorporation of glycan moieties through oxime formation. Moreover, the Derda group further showed the power of genetic incorporation to serve as an N-terminal functional handle as they demonstrated that 2-amino benzamidoxime (ABAO) derivatives could react with oxidized N-terminal serines present on the pIII protein of the phage (Kitov et al., 2014). This latter strategy can be used to functionalize the pIII minor coat proteins directly.

## CONCLUSION AND FUTURE DIRECTIONS

The chemical modification strategies described in this review showcase the current methods that have been used to create promising constructs; however, they also highlight many of the challenges of this field. Genetic modifications have allowed for the introduction of peptide motifs and natural amino acids that can act as handles for phage functionalization. In addition to the genetic incorporation of natural amino acids, unnatural amino acids have also been shown to be incorporated (Chen et al., 2007; Liu et al., 2008). With the new functional groups that this technique introduces comes a substantially expanded range of reactions that can be used to functionalize the phage by targeting uniquely reactive moieties such as ketones, azides, and alkynes. This will undoubtedly yield many selective modification strategies in the future. However, one major hurdle in using genetic approaches such as these is that the successes of these strategies are highly dependent on the ability of the phage coat proteins to tolerate genetic modifications.

The bioconjugation toolbox for phage functionalization is still limited in its ability to modify the scaffold under reaction conditions that will keep the construct functional. Commonly used bioconjugation techniques, such as NHS ester, EDC, and diazonium chemistry, have limited degrees of site-selectivity and can be difficult to control in terms of the modification they can achieve. If high levels of modifications are to be attempted, these chemical modifications can result in over-modification of the scaffold, rendering displayed peptides inactive and/or desolubilizing and destabilizing the construct. Site-selective bioconjugation strategies allow for the development of more well-defined constructs, which can obtain high levels of site-specific modification and still maintain function.

Although untested in the context of filamentous phage, a number functionalization strategies may prove to be effective tools in creating well-defined phage constructs. For example, enzymatic modifications such as those developed by Carrico et al. (2007) may allow for the incorporation of a chemical functional handle onto the phage scaffold. In addition to this, chemical strategies that target tyrosine residues could be applied, such as palladium pi-allyl chemistry (Tilley and Francis, 2006), Mannich reactions (Joshi et al., 2004), oxidative couplings (Seim et al., 2011), and a new tyrosine-click reaction (Ban et al., 2013). These strategies could expand the functional group tolerance beyond that of diazonium coupling reactions. Moreover, site-specific protein transamination using *N*-methylpyridinium-4-carboxaldehyde (Rapoport’s Salt) to introduce a carbonyl can be seen as a promising chemical modification strategy for the filamentous phage as the major coat protein pVIII contains a glutamate-rich sequence that is predicted to be reactive for this reagent (Witus et al., 2013). This strategy shows potential for achieving higher levels of modifications of the pVIII coat proteins that may be advantageous for a number of applications.

Despite the advances that have been made in phage functionalization, it is still generally difficult to construct scaffolds that can be modified in multiple locations in a well-defined manner. Future advances in the development of phage constructs will most likely focus on the development of combined genetic and chemical strategies that will allow for site-specific incorporation of multiple functionalities onto the virion. Although genetic engineering approaches have been shown to be powerful tools to display peptide functionalities on the phage scaffold, complementary chemical modification strategies can offer simple and reliable methods for appending widely varying functionalities to provide access to a wider variety of multifunctional materials.

## Conflict of Interest Statement

The authors declare that the research was conducted in the absence of any commercial or financial relationships that could be construed as a potential conflict of interest.
